# Myelin densities in retinotopically defined dorsal visual areas of the macaque

**DOI:** 10.1007/s00429-021-02363-z

**Published:** 2021-08-21

**Authors:** Xiaolian Li, Qi Zhu, Wim Vanduffel

**Affiliations:** 1 Laboratory for Neuro- and Psychophysiology, Department of Neurosciences, KU Leuven Medical School, 3000 Leuven, Belgium; 2 Cognitive Neuroimaging Unit, INSERM, CEA, Université Paris-Saclay, NeuroSpin Center, 91191 Gif/Yvette, France; 3 Leuven Brain Institute, KU Leuven, 3000 Leuven, Belgium; 4 Athinoula A. Martinos Center for Biomedical Imaging, Massachusetts General Hospital, Charlestown, MA 02129 USA; 5 Department of Radiology, Harvard Medical School, Boston, MA 02144 USA

**Keywords:** MRI, Myelination, Non-human primate, Visual cortex

## Abstract

The visuotopic organization of dorsal visual cortex rostral to area V2 in primates has been a longstanding source of controversy. Using sub-millimeter phase-encoded retinotopic fMRI mapping, we recently provided evidence for a surprisingly similar visuotopic organization in dorsal visual cortex of macaques compared to previously published maps in New world monkeys (Zhu and Vanduffel, Proc Natl Acad Sci USA 116:2306–2311, 2019). Although individual quadrant representations could be robustly delineated in that study, their grouping into hemifield representations remains a major challenge. Here, we combined in-vivo high-resolution myelin density mapping based on MR imaging (400 µm isotropic resolution) with fine-grained retinotopic fMRI to quantitatively compare myelin densities across retinotopically defined visual areas in macaques. Complementing previously documented differences in populational receptive-field (pRF) size and visual field signs, myelin densities of both quadrants of the dorsolateral posterior area (DLP) and area V3A are significantly different compared to dorsal and ventral area V3. Moreover, no differences in myelin density were observed between the two matching quadrants belonging to areas DLP, V3A, V1, V2 and V4, respectively. This was not the case, however, for the dorsal and ventral quadrants of area V3, which showed significant differences in MR-defined myelin densities, corroborating evidence of previous myelin staining studies. Interestingly, the pRF sizes and visual field signs of both quadrant representations in V3 are not different. Although myelin density correlates with curvature and anticorrelates with cortical thickness when measured across the entire cortex, exactly as in humans, the myelin density results in the visual areas cannot be explained by variability in cortical thickness and curvature between these areas. The present myelin density results largely support our previous model to group the two quadrants of DLP and V3A, rather than grouping DLP- with V3v into a single area VLP, or V3d with V3A+ into DM.

**Supplementary Information:**

The online version contains supplementary material available at 10.1007/s00429-021-02363-z.

## Introduction

**Fig. 1:**
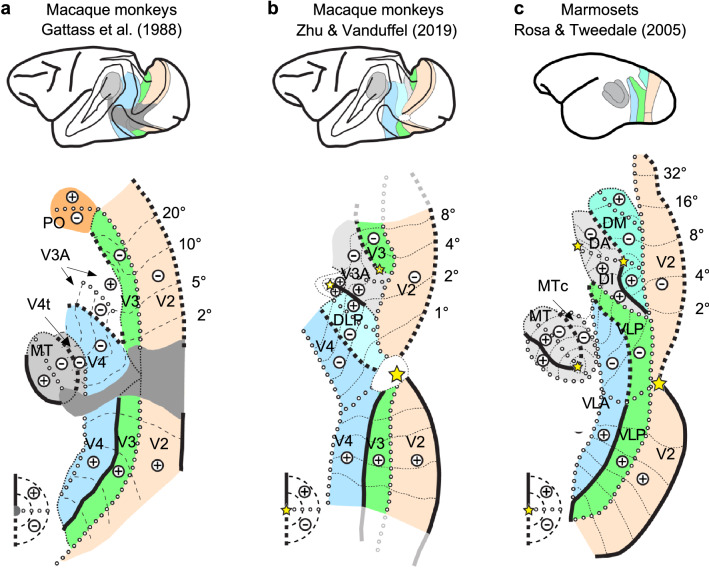
Different visuotopic models of early visual areas in Old and New World monkeys. **a** Most-widely accepted macaque model based on Gattass et al. (1988, 2015) [adapted from Rosa and Tweedale (2005)]. **b** New macaque model based on ultra-high-resolution retinotopic mapping (Zhu and Vanduffel 2019). **c** New World marmoset model based on Rosa and Tweedale (2005) and Angelucci and Rosa (2015) [adapted from Rosa and Tweedale (2005)]

The primate visual system encompasses more than 30 distinct visual areas (Felleman and Van Essen 1991). Characterizing the function and connectivity of each area, and the interactions between them, is a prerequisite to understand vision. This fundamental task, however, first requires a precise parcellation of visual cortex. Nonetheless, already at the early stages of the visual system, i.e., just rostral to the second visual area V2, the number and exact definition of third-tier visual areas are heavily contested, even after ~ 50 years since their initial discovery (Zeki 1969). This is, for example, evidenced by a specific volume devoted to this subject in the journal ‘Visual Neuroscience’ in 2015 (Gattass et al. 2015; Jeffs et al. 2015; Angelucci and Rosa 2015; Kaas et al. 2015; Sereno et al. 2015; Angelucci et al. 2015). The most prominent macaque model, which became textbook knowledge and served as the model for the layout of the human visual cortex, claims a single dorsal area V3 (V3d) stretching along the anterior border of dorsal area V2 (V2d) and representing at least the central 40° of the lower visual field (Fig. 1a). This simple but widely accepted model contrasts sharply with other models based on the functional topography observed in New World monkeys. Due to their lissencephalic brains, it is easier to discover fine-grained topographic functional properties in New World monkeys. Detailed research revealed that New World monkeys typically have a larger number of quadrant representations in dorsal visual cortex immediately rostral to V2d, even with multiple upper field quadrants within the swath of expected lower field representations (Fig. 1c). This contrasts sharply with only one upper field quadrant described in posterior dorsal visual cortex of the macaque monkey, which is typically assigned to area V3A (Fig. 1a).

The most likely source of these controversies relates to incomplete evidence supporting any of these models. The sequential nature and finite sample size of tractography and microelectrode recordings may have led to misinterpretations induced by uneven sampling and registration errors from 2D sections to 3D brains. fMRI, in contrast, has the advantage of revealing large-scale, detailed topographic information simultaneously across the entire brain. Several fMRI studies revealed detailed retinotopic maps encompassing the visual cortex of macaque monkeys (Vanduffel et al. 2002a; Brewer et al. 2002; Fize et al. 2003; Kolster et al. 2009, 2014; Janssens et al. 2014; Arcaro and Livingstone 2017). Most published fMRI results apparently confirm the widely accepted macaque model (Fig. 1a). However, fine-grained information may have been missed in these studies because of their relatively low spatial resolution.

To combat this resolution issue, we acquired phase-encoded retinotopic maps in awake macaque monkeys using implanted phased-array coils (Janssens et al. 2012) and contrast-agent enhanced fMRI at 3 T (Vanduffel et al. 2001). This yielded high-resolution maps (0.6 mm isotropic voxels), close in resolution to most microelectrode retinotopic mapping studies (0.3 ~ 0.5 mm in the z-direction, but 0.8 ~ 1 mm in the x–y plane). Using this technique, our most recent retinotopic mapping experiments (Zhu and Vanduffel 2019) revealed evidence for three areas instead of one, stretching along the rostral V2d border in macaques within 12° of eccentricity (Fig. 1b). Detailed population receptive field mapping, in combination with sub-mm field sign maps revealed that the two medial-most areas are third-tier areas. Only the most medial sector presumptively corresponds to ‘traditional’ area V3d, although reduced in its length (green colored area in Fig. 1b). The other middle sector corresponds to V3A as initially proposed by Zeki (Van Essen and Zeki 1978) (grey colored area in Fig. 1b). The most lateral area, dubbed DLP after its New World monkey equivalent (Sereno et al. 2015), is a fourth-tier area with a full hemifield representation (light blue colored area in Fig. 1b). In macaques, this area was either partly attributed to V3A and partly to the central portion of V3d (cfr Fig. 1a), or its two quadrants were assigned to two different areas based on New World monkeys (DI and a third-tier area VLP, cfr Fig. 1c). However, DLP fits better with our field sign and population receptive fields results, as well as with previous connectivity and electrophysiology evidence in macaques suggesting a second fourth-tier area in the prelunate gyrus (Zeki 1978; Maguire and Baizer 1984; Stepniewska and Kaas 1996; Youakim et al. 2001; Stepniewska et al. 2005).

The results of this high-resolution retinotopic mapping study suggest a new model that largely reconciles most reported discrepancies concerning the visuotopic organization of nonhuman primate caudo-dorsal occipital cortex. In this model, quadrants were mainly grouped based on visual field maps and population receptive fields, as measured with fMRI. Ideally, this type of information should be complemented with measures of other functional properties, connectivity, and cyto- and myelo-architectures (Yu et al. 2020). It is presently unknown, however, how the latter anatomical measurements would fit with the new model. Specifically, it is unknown what some of these anatomical properties are for the newly proposed area DLP in relation to V3d, V3v, V3A and V4. To address this question, in first instance without sacrificing the animals, and to further validate our model, we combined fine-grained retinotopic mapping with in-vivo measurements of myeloarchitectural features. In both humans and monkeys, myelin content has been visualized using MRI-based T1- and T2-weighted (T1- and T2-w) images and there is surprisingly good agreement between results obtained with such MR-defined myelin density maps and histologically obtained myelin measures (Bock et al. 2009; Glasser and Van Essen 2011; Lutti et al. 2014; Large et al. 2016). Even at mesoscopic scale, recent studies showed that variations in myelination across different stripe compartments of area V2 can be revealed using MR-defined high-resolution myelin density mapping in monkeys (Li et al. 2019) and humans (Dumoulin et al. 2017). Using a combination of high-resolution in-vivo myelin and retinotopic data, we quantitatively compared myelin densities in retinotopically defined visual areas of the macaque, partially acquired in the same individuals.

## Materials and methods

### Animals

High-resolution T1- and T2-w images were acquired from seven rhesus monkeys (Macaca mulatta; 4 males; 4–7 kg) to calculate myelin density maps, from which two subjects (M1 and M2) participated in our previous sub-millimeter retinotopic mapping study. Retinotopic maps from the third animal (M3) of our previous study, from which we have no myelin data, were also used in this study to create probabilistic retinotopic maps to define areas of interest in five new subjects in which only myelin density data were acquired. All animals were group-housed (cage size at least 16–32 m^3^) with cage enrichment (toys, foraging devices) at the primate facility of the KU Leuven Medical School. Animal housing and handling were according to the recommendations of the Weatherall report, allowing extensive locomotor behavior, social interactions, and foraging. Animal care and experimental procedures were performed in accordance with the National Institute of Health’s Guide for the Care and Use of Laboratory Animal, the European legislation (Directive 2010/63/EU) and were approved by the Ethical Committee of KU Leuven.

### MRI data acquisition

High-resolution (400 µm isotropic voxel size) T1- and T2-w images were acquired on a 3 T Siemens PRISMA scanner while the animals were under ketamine/xylazine anesthesia. A custom-built single loop coil with a diameter of 12 cm was used as receiver, and the body coil from the scanner was used for transmission. T1-w images were acquired using a magnetization prepared rapid gradient echo (MPRAGE) sequence (repetition time (TR) = 2700 ms, echo time (TE) = 3.5 ms, flip angle (*α*) = 9°, inversion time (TI) = 882 ms, matrix size 320 × 260 × 208) and T2-w images were acquired using a sampling perfection with application optimized contrasts using different flip angle evolution (SPACE) sequence (TR = 3200 ms, TE = 456 ms, variable *α*, matrix size 320 × 260 × 208, Turbo Factor = 131, echo spacing = 6 ms), as in (Van Essen et al. 2001; Glasser and Van Essen 2011). During one scan session, 7–12 T1-w images and 4–5 T2-w images were acquired from each subject, respectively.

### Data analysis

A surface representation of the cortex was first created for each subject using the averaged T1-w images. Image segmentation and surface creation was performed in Freesurfer following similar procedure as described for humans (Dale et al. 1999; Fischl et al. 1999). Myelin density maps were then calculated based on 400 µm isotropic resolution T1- and T2-w images acquired in the same session, following the procedure we described earlier (Li et al. 2019) and using the pipeline from Caret (Van Essen et al. 2001; Glasser and Van Essen 2011). The resulting myelin maps were smoothed using one iteration of Gaussian smoothing (strength = 1) and normalized into percentile scales within each hemisphere across the whole cortical surface area, to cancel out differences in absolute myelin density values across subjects, before data were pooled across hemispheres and subjects.

**Fig. 2:**
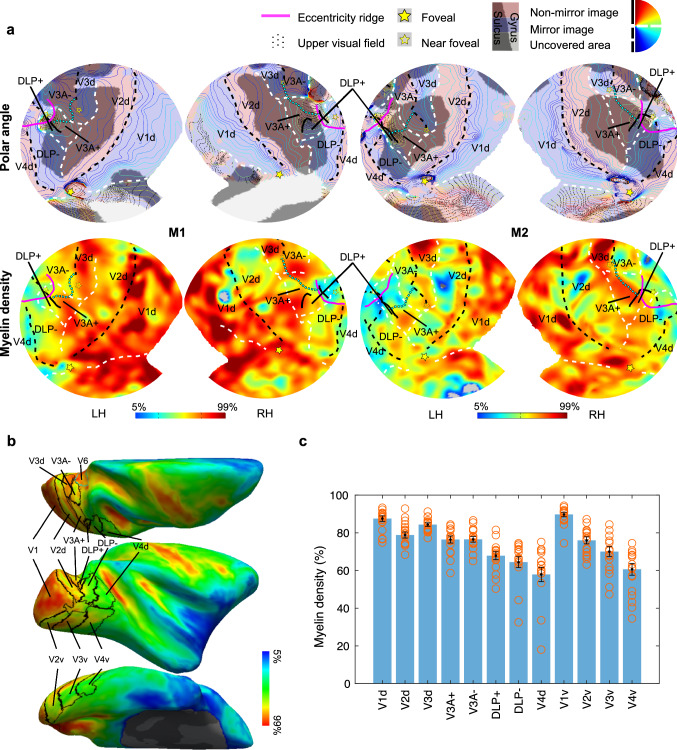
Myelin density variations across retinotopically defined visual areas. **a** Individual retinotopic (polar angle) and myelin density maps in M1 and M2 together with retinotopically defined areal borders. The cyan dashed line indicates the iso-eccentricity line that we followed to segregate the posterior and anterior portion of V3A. These two parts potentially correspond to marmoset DM and DI, respectively. **b** Median myelin density map presented on the right hemisphere of the common space (F99). Black lines indicate areal borders of the probabilistic map derived from six hemispheres of M1, M2 and M3. Cyan line indicates the areal border of V6 from Gamberini et al. (2015). **c** Mean myelin density estimates ($\pm$ standard error of the mean (SEM) across subjects) from the linear mixed-effect model. Orange circles indicate individual data points from all 14 hemispheres. LH, left hemisphere; RH, right hemisphere

To quantitatively compare myelin density across different areas, we extracted median myelin density (expressed in percentiles, for each hemisphere) across all voxels within each retinotopically defined area and compared these median densities across all regions of interest (ROIs) of all subjects. In M1 and M2, all ROIs were defined based on their own retinotopic maps. Each quadrant of V1, V2, V3, V3A, DLP and V4 in our model was defined as a single ROI. To test the alternative marmoset model (Fig. 1c), in a separate analysis, we split V3A into a posterior (potentially corresponding to marmoset DM) and an anterior part (presumptively corresponding to marmoset DI) of equal size along an iso-eccentricity line (cyan dashed lines in Fig. 2a). We kept all other ROIs the same in the latter analysis. In addition, to test the conventional macaque model (Fig. 1a), we split the lower visual field representation of DLP (DLP-) along an iso-eccentricity line into a central part that matches the width of V3d and a peripheral part which contains the remainder of DLP-. We directly compared the myelin density of central DLP- with that of V3d and peripheral DLP-. V2d was split similarly and used as a reference (Fig. 4). Since retinotopic maps were not available from the other five subjects of this study (M4-8), we created a probabilistic atlas from the three subjects (M1, M2 and M3) of our previous study in a common template surface space (F99), and used it to define probabilistic areas of interest for M4-8 (see Supplementary Fig. 1 for the alignment between the probabilistic ROIs and individually defined visual areas in M1-3). Retinotopic areas defined on the left hemispheres were projected to the right hemispheres using Freesurfer surface-to-surface registration tools before individual areas were aligned to the template. Ventral areas were also included in the analysis. Areas defined from the six hemispheres of M1-3 were pooled to create the probabilistic retinotopic atlas. A 50% probability threshold was applied for defining the probabilistic map. Myelin density maps (in percentiles) from the left hemispheres of M4-8 were also projected to the right hemispheres of the same subject to extract data from each area.

All 14 individual maps (2 hemispheres $\times$ 7 subjects) were aligned to the common template hemisphere to calculate the average map shown in Fig. 2b. Areal borders indicated using back lines correspond to the probabilistic retinotopic data derived from M1-3. To statistically compare myelin densities across all ROIs, median myelin density (in percentile) was calculated for each ROI in each hemisphere, and a linear mixed effect model with region as fixed effect, and subject and hemisphere as random effects, was fitted to the data from all ROIs. An false discovery rate (FDR) correction was used to account for multiple comparisons.

Finally, we quantified the influence of variability in both cortical thickness and folding pattern on our ROI-based myelin density analyses across areas and quadrants. First, we estimated whether a negative spatial correlation exists between cortical thickness and myelin density map, averaged across all hemispheres in the common space, as suggested based on human data (Glasser et al. 2014; Gomez et al. 2019). Individual cortical thickness maps were calculated in Freesurfer, and mapped onto the common surface space using the same surface-to-surface registration in Freesurfer as used for creating the myelin density maps. The thickness map from the left hemisphere was mapped to the right hemisphere in each individual before being mapped to the common space. All thickness maps of the individual hemispheres were then converted to percentiles before averaging across 14 hemispheres to create the average thickness map (Fig. 5a). Then Pearson correlation was used to calculate the spatial correlation between the average myelin density and cortical thickness map across the entire cortical sheet. In a second analysis, we calculated the median thickness (in percentile) in each ROI in each hemisphere, using the same procedures as the ROI-based myelin density analysis. We then calculated the correlation between cortical thickness and myelin density across all the ROIs across all hemispheres, to estimate how variability in cortical thickness is related to the observed myelin density differences. The same analyses were also conducted between cortical curvature and myelin density measurements to assess the influence of cortical folding variation across ROIs on the observed myelin density differences (Fig. 5b). Finally, to assess whether the ROI-based myelin density analysis was confounded by variability of cortical thickness and curvature across the ROIs, we regressed out cortical thickness and curvature variation across ROIs and hemispheres from the myelin density, and fed the residual into the ROI-based myelin density analysis.

## Results

**Fig. 3:**
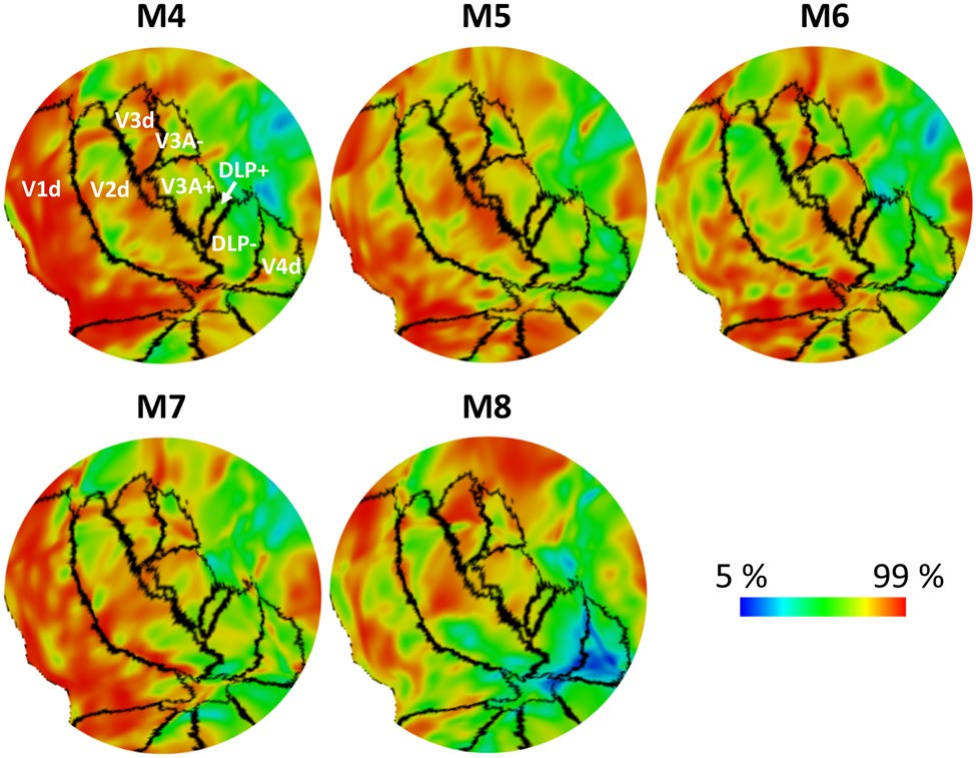
Myelin density variations across retinotopically defined visual areas are consistent among individual monkeys. Myelin density maps from both hemispheres in each subject are averaged and presented on the right hemisphere of the common space (F99). Black lines indicate areal borders of the probabilistic map derived from six hemispheres of M1, M2 and M3

The myelin density results from the four hemispheres of M1 and M2, together with their own retinotopic maps and retinotopically defined areal borders are shown in Fig. 2a. It is challenging to delineate areal borders based on the MRI-based myelin maps alone, since no sharp transitions in myelination density exist and high-resolution individual myelin maps revealed notable intra-areal heterogeneity of myelin density (see also Li et al. 2019). However, in conjunction with the retinotopically defined areal borders, some typical myelination features, and known intra-areal heterogeneity of myelin density as described earlier based on histological myelination staining, become apparent. For instance, V1 is highly myelinated in general, exactly as described previously (Donahue et al. 2018). V2 contains three main strip-like sub-compartments subserving different functions [for example, thin stripes are more color-biased, and thick stripes are more disparity- and motion-biased (Hubel and Livingstone 1987; Livingstone and Hubel 1987)], each showing different degrees of myelination [e.g., the pale stripes are more myelinated compared to the color- and disparity-/motion-biased stripes (Livingstone and Hubel 1984, 1988)]. Interestingly, in both M1 and M2, V2 shows alternating high and moderate myelinated compartments that run perpendicular to the V1/V2 border across the entire extent of V2, which correspond to functionally defined stripes as described in detail by Li et al. (2019). In both subjects, these compartments seem to extend further into the third-tier areas, corroborating histology and electrophysiological evidence for alternating dark and light CO bands (Vanduffel et al. 2002b; Sincich et al. 2003) and disparity-selective columns in V3 of macaques (Adams and Zeki 2001), and recent human fMRI evidence showing similar interdigitated color- and disparity-/motion-selective stripes within area V3 (Nasr et al. 2016; Dumoulin et al. 2017). Area V3d, as defined by our high resolution retinotopic mapping, also appears to be highly myelinated. Together with the posterior portion of V3A, which is segregated from its anterior portion by the cyan dashed line, they form a heavily myelinated zone immediately rostral to V2d, very similar to DM as described in the marmoset (Rosa and Tweedale 2000). In some hemispheres, this heavy myelination band extends further into the central portion of DLP, forming a narrow band that resembles the elongated V3d as proposed in the conventional macaque model (Fig. 1a). Qualitatively, however, V3d also appears to show higher myelin densities compared to both the posterior portion of V3A, and DLP. More medially, this highly myelinated band extends further into the mesial surface of the hemisphere and to the anterior bank of the parieto-occipital sulcus (Fig. 2b). This section corresponds to an area named the parieto-occipital area (PO) (Lewis and van Essen 2000) or V6 (Gamberini et al. 2015; Hadjidimitrakis et al. 2019), which was not identified in our retinotopic experiment due to limited visual field coverage of the stimuli (up to 12° of eccentricity). All these above-mentioned myelination features are also presented in the other five subjects (M4-8), as shown in Fig. 3.

To quantitatively compare myelin densities across different areas defined by our retinotopic experiment, we extracted median myelin density values (in percentiles, for each hemisphere) within each ROI and compared them across all ROIs of all subjects. The result showed a main effect of region (*P* < 10^–11^), indicating that retinotopically defined areas of dorsal visual cortex have different myelin densities. A post hoc pairwise comparison further revealed that myelin densities are higher in V3d and both quadrants of V3A compared to more lateral areas (i.e., DLP-, *Ps* < 0.01, FDR corrected) (statistical values between all possible region pairs are also included in Table 1). Unlike the marmoset (Rosa and Tweedale 2000), myelin density in V3d appears to be significantly higher than V3A+ (*P* < 0.05, FDR corrected). A significant difference was also observed between V3d and the two portions of V3A, when V3A was segregated into a posterior and anterior portion (*P* < 0.02, FDR corrected). Importantly, myelin densities are indistinguishable between V3A- and V3A+ , and also between the posterior and anterior divisions of V3A (*P*s > 0.9, uncorrected). Moreover, myelin density is significantly higher in V3d compared to V3v (*P* < 0.05, FDR corrected) (Fig. 2c), even when the V3v ROI was restricted to match the eccentricity coverage of V3d (*P* < 0.01, FDR corrected), consistent with the observations of Van Essen and colleagues (Burkhalter and Van Essen 1986; Felleman and Van Essen 1987). However, opposite to the qualitative observation in marmosets (Rosa and Tweedale 2000), we observed a significantly higher myelination in V3v compared to DLP- (*P* < 0.05, FDR corrected). Myelin densities are not distinguishable between dorsal and ventral portions of V1, V2 and V4, respectively, and between V2 and V3A. Area V4 shows significantly lower myelination density compared to both quadrants of V2, V3 and V3A. Finally, myelin densities in DLP- cannot be distinguished from DLP+ (*P* = 0.2, uncorrected).

**Table 1:** Post hoc pairwise comparison of myelin densities between all possible region pairs (two-tailed *t* test)^a^

	V1d	V2d	V3d	V3A+	V3A−	DLP+	DLP−	V4d	V1v	V2v	V3v	V4v
V1d	–	0.0013 (0.0022)	0.2371 (0.2746)	0.0001 (0.0001)	0.0001 (0.0001)	0.0000 (0.0000)	0.0000 (0.0000)	0.0000 (0.0000)	0.4119 (0.4385)	0.0000 (0.0001)	0.0000 (0.0000)	0.0000 (0.0000)
V2d	–	–	0.0394 (0.0490)	0.3756 (0.4132)	0.3922 (0.4244)	0.0001 (0.0001)	0.0000 (0.0000)	0.0000 (0.0000)	0.0001 (0.0001)	0.2900 (0.3300)	0.0012 (0.0020)	0.0000 (0.0000)
V3d	–	–	–	0.0035 (0.0051)	0.0038 (0.0055)	0.0000 (0.0000)	0.0000 (0.0000)	0.0000 (0.0000)	0.0462 (0.0565)	0.0020 (0.0031)	0.0000 (0.0000)	0.0000 (0.0000)
V3A+	–	–	–	–	0.9756 (0.9757)	0.0015 (0.0024)	0.0000 (0.0000)	0.0000 (0.0000)	0.0000 (0.0000)	0.8628 (0.8760)	0.0168 (0.0222)	0.0000 (0.0000)
V3A−	–	–	–	–	–	0.0014 (0.0022)	0.0000 (0.0000)	0.0000 (0.0000)	0.0000 (0.0000)	0.8388 (0.8651)	0.0155 (0.0209)	0.0000 (0.0000)
DLP+	–	–	–	–	–	–	0.2013 (0.2372)	0.0003 (0.0005)	0.0000 (0.0000)	0.0027 (0.0040)	0.4203 (0.4403)	0.0076 (0.0106)
DLP−	–	–	–	–	–	–	–	0.0153 (0.0209)	0.0000 (0.0000)	0.0000 (0.0001)	0.0381 (0.0484)	0.1568 (0.1881)
V4d	–	–	–	–	–	–	–	–	0.0000 (0.0000)	0.0000 (0.0000)	0.0000 (0.0000)	0.3049 (0.3411)
V1v	–	–	–	–	–	–	–	–	–	0.0000 (0.0000)	0.0000 (0.0000)	0.0000 (0.0000)
V2v	–	–	–	–	–	–	–	–	–	–	0.0262 (0.0339)	0.0000 (0.0000)
V3v	–	–	–	–	–	–	–	–	–	–	–	0.0006 (0.0010)

Values in each cell are the uncorrected and FDR corrected (in brackets) *P* values

**Fig. 4:**
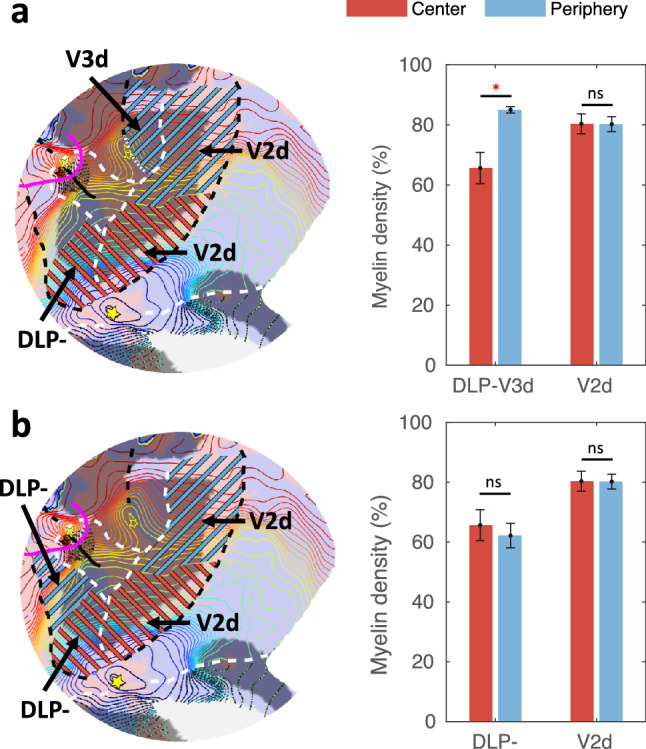
Myelin densities in central DLP- compared with its peripheral counterparts (V3d and DLP-). **a** Myelin density in central DLP- and V2d (red hatching in left panel) compared with their peripheral counterparts (V3d and V2d) (blue hatching in left panel). **b** Myelin density in central DLP- and V2d (red hatching in left panel) compared with their peripheral counterparts (DLP- and V2d) (blue hatching in left panel). *P < 0.01; ns, *P* > 0.05; Bars and error bars, mean and SEM across subjects

Next, in the conventional macaque model (Fig. 1a), the central part of DLP- belongs to V3d, whereas its peripheral part belongs to a different area, i.e., area V3A. To directly test myelin density differences based on this model, we split DLP- along an iso-eccentricity line into a central and a peripheral portion (see methods) and directly compared the myelin density of central DLP- with that of V3d and peripheral DLP-. V2d was split similarly and used as a reference. As shown in Fig. 4, the myelin density in V3d is significantly higher compared to both the central and peripheral portions of DLP- (*P*s < 10^–3^, FDR corrected) (top panel, Fig. 4), yet myelin densities of the two DLP sectors are indistinguishable (*P* = 0.2416, uncorrected) (bottom panel, Fig. 4), exactly as observed in the central and peripheral portions of V2d (*P* = 0.6292, uncorrected). Moreover, there is a significant region $\times$ visual field interaction when central DLP was grouped with V3d (*P* < 10^–3^, Fig. 4, top panel). This is not the case, however, when central DLP was grouped with its peripheral part (*P* = 0.425, Fig. 4, bottom panel) and compared with central and peripheral V2d.

**Fig. 5:**
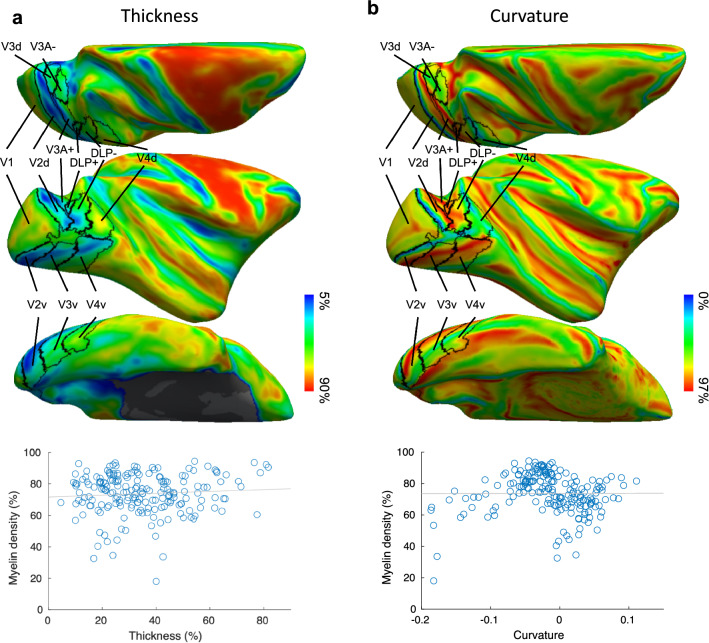
Cortical thickness and folding pattern variations across retinotopically defined visual areas. **a** Average cortical thickness map presented on the right hemisphere of the common space (F99) (upper panel) and the correlation between median cortical thickness and myelin density across all the ROIs and hemispheres (lower panel). Each blue dot represents the median cortical thickness and myelin density in one ROI in one individual hemisphere. **b** Average cortical curvature map presented on the right hemisphere of the common space (F99) (upper panel) and the correlation between cortical curvature and myelin content across all the ROIs and hemispheres (lower panel). Each blue dot represents the median cortical curvature and myelin density in one ROI in one individual hemisphere. Black lines in upper panels indicate areal borders of the probabilistic map derived from six hemispheres of M1, M2 and M3

Finally, based on human MRI data it has been suggested that a negative correlation exists between cortical thickness and myelin content, calculated as an average across all the cortical layers in proportion to their volume (Glasser et al. 2014; Gomez et al. 2019). In this study, we adopted the same approach to estimate myelin density and we also obtained the same negative correlation when the averaged (across the 14 hemispheres) cortical thickness and myelin densities were correlated across the entire cortical sheet (*r* = −0.302, *P* < 0.001). However, when we calculated the median myelin density within the areas of interest, and correlated these values with the corresponding mean cortical thicknesses across all the data points, we did not find a significant correlation between these two indices (*r* = 0.0758, *P* = 0.296, Fig. 5). We also performed the same analysis between myelin density and cortical curvature and obtained the same results: there is a significant positive correlation between myelin density and curvature across the entire cortical sheet (*r* = 0.127, *P* < 0.001), but there is no correlation between median myelin density and curvature across all the ROIs and hemispheres (*r* = 0.003, *P* = 0.96, Fig. 5). Moreover, the overall areal differences in myelin density did not change after regressing out the median cortical thickness and curvature across all the data points (see Supplementary Table 1). These results suggest that the observed areal difference in myelin density is not confounded by differences in both cortical thickness and folding patterns across the areas and visual field quadrants studied.

## Discussion

The present results largely support our proposed model of posterior dorsal visual cortex based on high resolution retinotopy data (Zhu and Vanduffel 2019). In this model, we grouped the two quadrants of DLP and those of V3A, rather than combining V3d with V3A+ , or the posterior part of V3A into a single area DM, or yet alternatively DLP- with V3v into a putative VLP. Consistent with this model, V3d and V3A+ (or the posterior portion of V3A) not only differ in visual field sign and pRF size, but also in myelin density. Therefore, at least based on current (f)MRI-based observations, it is unreasonable to combine them into a single area. Instead, V3A- and V3A+ are indistinguishable when considering all three measures (pRF, field sign and myelin density), hence these are most likely two quadrants belonging to a single area (i.e., V3A). Based on visual field representations, V3v could theoretically be combined with DLP- to form a complete representation of the visual field, as VLP in New World monkeys (Fig. 1c). However, these two quadrants show significantly different pRF sizes and myelin densities. Furthermore, no significant difference in myelin density was observed between DLP+ and DLP-. Therefore, the most parsimonious combination of quadrants is DLP- with DLP+ , rather than DLP- with V3v, exactly as we proposed earlier (Zhu and Vanduffel 2019). This leaves V3d as the only remaining quadrant that can be combined with V3v to form a complete hemifield representation.

Indeed, among the three measures (pRF, field sign and myelin density), V3d only differs from V3v by its higher myelination density, as also reported in earlier histological myelin staining studies (Burkhalter and Van Essen 1986; Felleman and Van Essen 1987). If more weight is given to the visual field representation and pRF size, they could be considered parts of the same area, as proposed traditionally (Gattass et al. 1988). It needs to be noted that a similar ‘within-area’ myelin density asymmetry has been also described for area MT, in which myelin density drops at more peripheral visual field representations (Allman and Kaas 1971; Fiorani et al. 1989; Sereno et al. 2015). Area V2 is characterized by its different stripe compartments, which show different degrees of myelination (Tootell et al. 1983; Horton and Hocking 1997; Dumoulin et al. 2017). Hence it is reasonable to assume that this is also the case for V3d and V3v. Alternatively, V3d and V3v could be considered parts of different areas. Indeed, early studies have shown that V3/V3d and VP/V3v differ not only in myelo-architecture, but also in functional properties. For example, V3d contains more direction-selective, yet fewer color-selective neurons compared to VP/V3v (Burkhalter et al. 1986; Burkhalter and Van Essen 1986; Felleman and Van Essen 1987; Felleman et al. 1997). Although still debated, different connectivity patterns have also been observed between V3d and V3v. Some studies observed afferents from V1 projecting towards V3/V3d but not VP/V3v (Burkhalter et al. 1986; Felleman et al. 1997). Another study using more sensitive tracers, however, also observed afferent projections from V1 towards VP/V3v (Lyon and Kaas 2002). Yet, it is still an open question whether the V1 projections to VP/V3v and V3/V3d arise predominantly from the same layer (i.e., layer 4). Although V3/V3d is different from VP/V3v based on its denser myelination pattern and functions (more direction-selective and less color-selective neurons), it is counterintuitive (or improbable) to consider them as separate single areas since each of these areas would contain only one quadrant representation (Zeki 2003).

In summary, although it remains challenging to conclude whether V3d and V3v belong to the same area, our current evidence largely supports our previous model to group the two quadrants of DLP and V3A, rather than grouping DLP- with V3v into a single area VLP, or V3d with V3A+ into a putative DM. Importantly, our myelin density results are not confounded by differences in cortical thickness and folding patterns across these areas and quadrants. Our analyses are limited to the cortex representing the central 12° of eccentricity. Large-field retinotopic mapping is still required to better understand the functional organization of visual cortex representing higher eccentricities (Gamberini et al. 2015; Hadjidimitrakis et al. 2019; Rima et al. 2020). Future studies combining ultra-high-resolution functional imaging and MR-based histology (Eickhoff et al. 2005; Xu et al. 2019; Assaf 2019) in the same subjects will also provide a more comprehensive picture of mesoscale cortical variations across layers, ultimately leading to a comprehensive parcellation of primate visual cortex.

## Supplementary Information

Below is the link to the electronic supplementary material.

Supplementary file1 (PDF 1491 KB)

Supplementary file2 (PDF 93 KB)

## Declarations

### Availability of data and material

Data are available from the corresponding authors upon reasonable request.

### Code availability

NA
