# Supplementary material for: Myelin densities in retinotopically defined dorsal visual areas of the macaque
Source: Brain Struct Funct. 2021 Aug 21;226(9):2869–80. doi: 10.1007/s00429-021-02363-z (PMC8541961; doi:10.1007/s00429-021-02363-z)
Supplement: Supplementary file 1 — Supplementary file1 (PDF 1491 KB) [file 429_2021_2363_MOESM1_ESM.pdf]

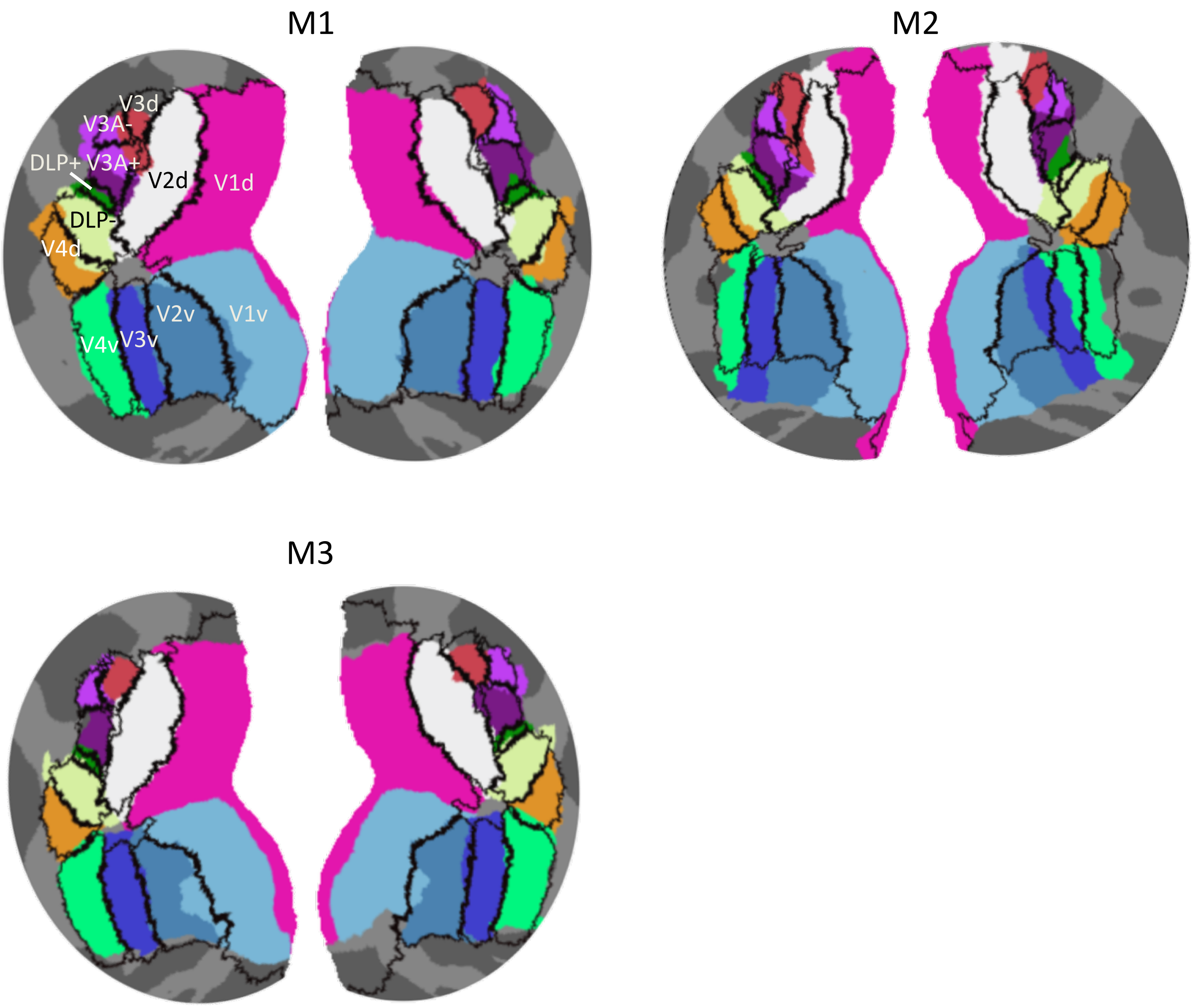

Supplementary Fig. 1 Individually defined visual areas overlapped with areal borders (black lines) of the probabilistic map derived from the 6 hemispheres of M1, M2 and M3. Retinotopically defined visual areas are indicated with different colors as shown in the left hemisphere in M1.
