# Supplementary material for: Myelin densities in retinotopically defined dorsal visual areas of the macaque
Source: Brain Struct Funct. 2021 Aug 21;226(9):2869–80. doi: 10.1007/s00429-021-02363-z (PMC8541961; doi:10.1007/s00429-021-02363-z)
Supplement: Supplementary file 2 — Supplementary file2 (PDF 93 KB) [file 429_2021_2363_MOESM2_ESM.pdf]

Supplementary Table 1 Post-hoc pairwise comparison of myelin densities between all possible region pairs (two-tailed *t*-test)[a] after regressing out cortical thickness and curvature variation across ROIs and hemispheres

	V1d	V2d	V3d	V3A+	V3A-	DLP+	DLP-	V4d	V1v	V2v	V3v	V4v
V1d	-	0.0108 (0.0146)	0.4379 (0.4588)	0.0002 (0.0003)	0.0002 (0.0003)	0.0000 (0.0000)	0.0000 (0.0000)	0.0000 (0.0000)	0.3606 (0.3839)	0.0003 (0.0006)	0.0000 (0.0000)	0.0000 (0.0000)
V2d	-	-	0.0732 (0.0912)	0.1943 (0.2290)	0.2092 (0.2422)	0.0000 (0.0000)	0.0000 (0.0000)	0.0000 (0.0000)	0.0006 (0.0010)	0.2817 (0.3136)	0.0003 (0.0005)	0.0000 (0.0000)
V3d	-	-	-	0.0022 (0.0034)	0.0026 (0.0039)	0.0000 (0.0000)	0.0000 (0.0000)	0.0000 (0.0000)	0.0921 (0.1126)	0.0045 (0.0066)	0.0000 (0.0000)	0.0000 (0.0000)
V3A+	-	-	-	-	0.9662 (0.9662)	0.0008 (0.0012)	0.0000 (0.0000)	0.0000 (0.0000)	0.0000 (0.0000)	0.8237 (0.8495)	0.0166 (0.0215)	0.0000 (0.0000)
V3A-	-	-	-	-	-	0.0007 (0.0011)	0.0000 (0.0000)	0.0000 (0.0000)	0.0000 (0.0000)	0.8569 (0.8701)	0.0149 (0.0196)	0.0000 (0.0000)
DLP+	-	-	-	-	-	-	0.2403 (0.2734)	0.0002 (0.0005)	0.0000 (0.0000)	0.0004 (0.0006)	0.3147 (0.3405)	0.0080 (0.0115)
DLP-	-	-	-	-	-	-	-	0.0108 (0.0146)	0.0000 (0.0000)	0.0000 (0.0000)	0.0302 (0.0383)	0.1340 (0.1608)
V4d	-	-	-	-	-	-	-	-	0.0000 (0.0000)	0.0000 (0.0000)	0.0000 (0.0000)	0.2851 (0.3136)
V1v	-	-	-	-	-	-	-	-	-	0.0000 (0.0000)	0.0000 (0.0000)	0.0000 (0.0000)
V2v	-	-	-	-	-	-	-	-	-	-	0.0090 (0.0127)	0.0000 (0.0000)
V3v	-	-	-	-	-	-	-	-	-	-	-	0.0003 (0.0005)

a. Values in each cell are the uncorrected and FDR corrected (in brackets) *P* values.
